# Effectiveness of school-based interventions for preventing tobacco smoking initiation among young people in low- and middle-income countries: a systematic review protocol

**DOI:** 10.1186/s13643-022-02127-8

**Published:** 2022-11-23

**Authors:** Divine Darlington Logo, Yeetey Enuameh, George Adjei, Arti Singh, Emmanuel Nakua, Edward Dassah, Felix Boakye Oppong, Ellis Owusu-Dabo

**Affiliations:** 1 Ghana Health Service, Research and Development Division, Accra, Ghana; 2 Department of Global and International Health, School of Public Health, College of Health Sciences, Kwame Nkrumah University of Science and Technology, Kumasi, Ghana; 3 Department of Epidemiology and Biostatistics, School of Public Health, College of Health Sciences, Kwame Nkrumah University of Science and Technology, Kumasi, Ghana; 4 Department of Community Medicine, School of Medical Sciences, College of Health and Allied Science, University of Cape Coast, Cape Coast, Ghana; 5 Department of Population and Reproductive Health, School of Public Health, College of Health Sciences, Kwame Nkrumah University of Science and Technology, Kumasi, Ghana; 6 Global Statistical Institute, Global Statistical Institute, Techiman, Ghana; 7 JBI Center of Excellence: Kintampo Health Research Center, Kintampo, Ghana

**Keywords:** Smoking, School-based intervention, Education, Smoking initiation prevention, Randomized, Youth, Students

## Abstract

**Background:**

Despite the commendable progress made globally in tobacco control, the world is falling short of achieving a 30% relative reduction in current tobacco use by 2025. The African region remains the least in the efforts in fighting the tobacco epidemic and is most exploited by the tobacco industry. Schools have been continuously used for over three decades as a setting for delivering youth smoking prevention programmes; however, the evidence of the effectiveness of those school-based interventions provides varying outcomes. Also, interventions that proved to be effective, in high-income countries (HICs), may not necessarily be effective in the African region as a result of cultural differences and other contrasting factors.

An existing systematic review that explored school-based tobacco prevention programmes among the youth in African countries from 2000 to 2016 showed partial effectiveness. This review will address the gap by updating the 2016 review to examine studies in LMICs to generate findings to help target resources which have the potential to save lives by preventing smoking initiation among young people.

**Methods:**

The JBI methodology for systematic reviews of effectiveness will guide the conduct of this review. A comprehensive strategic search will be developed to retrieve both published and unpublished studies that evaluate school-based interventions to prevent tobacco smoking initiation among in-school young people in LMICs compared to non-intervention programmes. Published studies would be from databases such as MEDLINE via Ovid, CINAHL via EBSCO, Embase, PsycINFO, PsycEXTRA, and the Cochrane Central Register of Controlled Trials. Sources of grey literature would be ProQuest Dissertations and Theses, MedNar, EBSCO Open Dissertations, Open Access Theses and Dissertations, and Trove.

The databases will be searched for published studies in the English language. The processes of study selection, critical appraisal, data extraction, and data synthesis will be in accordance with the JBI approach for reviews of effectiveness with a minimum of two reviewers at each stage. The primary outcome of the review will be the non-initiation of tobacco smoking by the youth.

**Discussion:**

The review will provide synthesized evidence on the effectiveness of school-based smoking initiation prevention among young people in LMICs. The findings of the review would support policymakers and programme implementers to develop targeted interventions for effective tobacco control initiatives.

**Systematic review registration:**

PROSPERO CRD42021246206

**Supplementary Information:**

The online version contains supplementary material available at 10.1186/s13643-022-02127-8.

## Background

The use and exposure to tobacco smoke are associated with increased morbidity and mortality among adolescents, contributing to a rise in asthma, bronchitis, tuberculosis, inflammatory bowel disease, and cancers worldwide [1]. However, the trend of smoking rate is considerably declining in developed countries, while the reverse holds for the African region [2]. It is suggested most tobacco-related diseases will affect the African region because over 80% of the world’s tobacco users live in low- and middle-income countries including the African region [3].

According to the World Health Organization (WHO) global report on “trends in the prevalence of tobacco use from 2000-2025”, the world will fall short of achieving a 30% relative reduction in current tobacco use by the year 2025, even though there is commendable progress made in tobacco control globally [4]. Globally, adolescents’ tobacco use varies from 2% to over 30% depending on the area, where about a fifth of young people are smokers [5]. Low- and middle-income countries (LMICs) have higher rates of adolescent smoking, particularly among boys where some countries report high as 46% of tobacco use among the youth [5].

The African region is reported to be the least in the fight against tobacco epidemics [4]. The region has a prevalence of current use of any tobacco product among adolescents of 19.1% (males — 23.7%; females — 13.7%), but Zimbabwe recorded the highest at 47.1% and Morocco at 12.6%, respectively [6].

Given that most smoking initiation starts in adolescence, there has been a series of interventions developed globally to prevent initiation and/or reduce tobacco consumption and the associated negative consequences. Therefore, over the past three decades, the school environment has been a particular focus of efforts to influence youth smoking behaviour [7]. The main perceived advantages are that almost all children can be reached through schools, and a focus on tobacco education fits naturally within their daily activities. Hence, several interventions targeting adolescents were developed, including mass media campaigns, tobacco cessation and treatment, smoke-free laws, price and tax measures, and minimum age restrictions [8]. These school-based tobacco prevention programmes are considered cost-effective strategies for reducing smoking prevalence among young people [9]. These programmes facilitate skill building, problem-solving skills, decision-making to avoid tobacco use, and coping strategies for adolescents who are already smokers and are addicted to staying away from tobacco use [10].

Even though previous school-based smoking prevention studies showed varying results, some interventions had been associated with increased knowledge, positive attitude, and behaviour toward smoking [11] and reduced smoking initiation [12] among young people. Nishio and colleagues [13] systematic review found that school-based intervention programmes in preventing tobacco use in the African region among young people had positive effects on improving knowledge of the harmful effects of tobacco use and attitudes toward smoking initiation and reduced smoking cessation. The team, however, could not establish any statistical significance of the intervention in reducing smoking prevalence among African students [13].

It has been observed that effective health interventions based in the context of HICs are not necessarily effective in LMICs [14, 15]; therefore, there is the need to identify and review interventions conducted in LMICs including Africa for effective policies in tobacco control, especially among the youth.

This systematic review will address the gap by updating the 2016 review to examine current school-based intervention programmes in LMICs including Africa. This is aimed to generate findings to help target resources which have the potential to save lives by preventing smoking initiation among young people.

## Method/design

The proposed systematic review will be conducted in line with the JBI methodology for systematic reviews of effectiveness (27). We will also use the Preferred Reporting Items for Systematic review and Meta-Analyses (PRISMA) guidelines and its extension for protocols (PRISMA-P) (28) (see Additional file 1: PRISMA-P checklist).

### Research objective

This systematic review aims to evaluate the effectiveness of school-based interventions compared to non-intervention programmes in preventing tobacco smoking initiation among young people in LMICs.

Specifically, we will do the following:

1. Assess the effects of school-based interventions in preventing smoking initiation among young ones.
2. Assess the effects of school-based interventions in reducing the smoking rate (reduction in the number of cigarettes smoked).

### Review question

What is the effectiveness of school-based programmes classified by intervention type compared to no intervention in preventing tobacco smoking initiation among young people living in LMICs?

### Inclusion criteria

The systematic review will consider studies that evaluate school-based interventions that prevent tobacco smoking initiation among in-school young people in LMICs compared to non-intervention programmes. The primary outcome will be the non-initiation of tobacco smoking by the youth. Studies published in the English language will be considered for the review to align with current trends in tobacco intervention programmes.

#### Participants

This review will consider studies that include in-school persons aged 10–24 years in LMICs.

#### Interventions

The review will consider studies that evaluate school-based interventions that aim at preventing tobacco smoking initiation or tobacco use among young people who reside in LMICs. The types of interventions based on theoretical approaches such as only information on harmful effects of tobacco use, social influence, social competence, a combination of social influence, and social competence or multimodal, i.e. a combination of information, social influence, and competence, will be considered. Furthermore, the approach to the administration of the intervention, i.e. teacher led, peer led, or researcher led, will be considered. Finally, the presence or absence of booster sessions will be considered.

#### Comparator

The review will consider studies that compare the intervention with non-intervention such as normal school education or the standard health education programmes taught in schools.

#### Outcomes

The review will consider studies whose outcome evaluates the smoking status of individuals or groups who reported tobacco non-use at baseline. The theoretical approach, mode of administration of interventions, and the presence or absence of booster sessions will be considered.

Effect sizes of the outcome would be measured by the rate/risk difference, rate/risk/odds ratio for categorical data, and weighted/standardized mean difference for continuous data together with their 95% confidence intervals.

#### Type of studies

This review will consider both experimental and quasi-experimental study designs including randomized controlled trials, non-randomized controlled trials, and before and after studies. Databases will be searched for all studies published in peer-reviewed journals in the English language and will be included in the review.

#### Search strategy

The search strategy will aim to locate both published and unpublished studies. An initial limited search of MEDLINE and CINAHL was undertaken to identify articles on the topic. The text words contained in the titles and abstracts of relevant articles, and the index terms used to describe the articles were used to develop a full search strategy for MEDLINE (PubMed, see Appendix). The search strategy, including all identified keywords and index terms, will be adapted for each included information source (database). The reference list of all studies selected for critical appraisal will be screened for additional studies. The following keywords would be used: young people, tobacco use, school-based tobacco prevention programme, LMICs, and Africa.

#### Information sources

The databases to be searched include MEDLINE via Ovid, CINAHL via EBSCO, Embase, PsycINFO, PsycEXTRA, and the Cochrane Central Register of Controlled Trials. Sources of grey literature to be searched include ProQuest Dissertations and Theses, MedNar, EBSCO Open Dissertations, Open Access Theses and Dissertations, and Trove.

#### Data management and study selection

Following the search, all identified citations will be ordered and uploaded into Mendeley [16], and duplicates removed. Titles and abstracts will then be screened by two independent reviewers for assessment against the inclusion criteria for the review. Relevant studies will be retrieved and saved in full and their citation details imported into the JBI System for the Unified Management, Assessment, and Review of Information (JBI SUMARI; JBI, Adelaide, Australia) [17]. The full text of selected citations will be assessed in detail against the inclusion criteria by two independent reviewers. Reasons for the exclusion of full-text studies that do not meet the inclusion criteria will be recorded and reported in the systematic review. Any disagreements that arise between the reviewers at each stage of the study selection process will be resolved through discussion or with a third reviewer. The results of the search will be reported in full in the final systematic review and presented in the Preferred Reporting Items for Systematic Reviews and Meta-Analyses (PRISMA) flow diagram [18].

#### Assessment of methodological quality

Eligible studies will be critically appraised by two independent reviewers at the study level for methodological quality in the review using a standardized critical appraisal tool from the JBI for experimental, quasi-experimental, and analytical observational studies [19]. Authors of papers will be contacted to request missing or additional data for clarification, where required. Any disagreements that arise will be resolved through discussion or with a third reviewer. The results of the critical appraisal will be reported in narrative form and a table. Cut-off scores will not be employed as all studies, regardless of the results of their methodological quality, and will undergo data extraction and synthesis (where possible). Critical appraisal scores would be employed in assessing the certainty of findings using the Grading of Recommendations, Assessment, Development and Evaluation (GRADE) approach.

#### Data extraction

Data will be extracted from studies included in the review by two independent reviewers using the standardized data extraction tool within the JBI SUMARI [20]. The data extracted will include specific details about the populations, study methods, interventions, intervention, and control/comparison groups and outcomes (incidence/rate/odds ratios, incidence/rate difference, weighted/standardized mean differences, etc.) of significance to the review objective. Any disagreements that arise between the reviewers will be resolved through discussion or with a third reviewer. Authors of papers will be contacted to request missing or additional data, where required.

#### Data synthesis

Studies will, where possible be pooled in a statistical meta-analysis using JBI SUMARI. Effect sizes will be expressed as either odds/rate/risk ratios or incidence/rate differences (for dichotomous data) and weighted (or standardized) final post-intervention mean differences (for continuous data) and their 95% confidence intervals will be calculated for analysis. Heterogeneity will be assessed statistically using the standard chi-squared (Cochran’s Q test) and I-squared tests.

Statistical analyses will be performed using a random-effects model [21]. Sensitivity and subgroup analyses will be conducted for 10 or more studies to assess the robustness of the methods used. Where statistical pooling is not possible the findings will be presented in a narrative form including tables and figures to aid in data presentation where appropriate. A funnel plot will be generated using RevMan V5 (Copenhagen: The Nordic Cochrane Centre, Cochrane) to assess publication bias if there are 10 or more studies included in a meta-analysis. Statistical tests for funnel plot asymmetry (Egger test, Begg test, Harbord test) will be performed where appropriate.

#### Assessing certainty in findings

The Grading of Recommendations, Assessment, Development and Evaluation (GRADE) approach for grading the certainty of evidence will be followed [22], and a summary of findings (SoF) will be created using GRADEPro GDT Version XX/updated in 2014 (McMaster University, ON, Canada) [11]. The SoF will present the following information where appropriate: absolute risks for the treatment and control, estimates of relative risk, and a ranking of the quality of the evidence based on the risk of bias, directness, heterogeneity, precision, and risk of publication bias of the review results. The outcomes to be reported in the SoF will be the non-initiation of tobacco smoking, the theoretical approaches employed in the intervention, the mode of administration of interventions, and the presence or absence of booster sessions.

## Discussion

This systematic review will generate evidence on the effectiveness of school-based interventions regarding smoking initiation and quit rates. Evidence will further be generated on the effectiveness of interventions based on their theoretical bases and mode of programme delivery in LMICs. Findings will help policymakers and programme implementers to develop targeted interventions for effective tobacco control initiatives. This review will generate evidence to inform the design of future interventions in schools to prevent children and young people from smoking in LMICs. This could help target resources appropriately and has the potential to save lives by preventing smoking initiation among children and young people. There are some limitations to the outlined systematic review. The restriction to English is acknowledged as a language bias. The cost of high-quality translations is beyond the resources of this review.

### Supplementary Information

**Additional file 1.** PRISMA-P 2015 Checklist

## Data Availability

Not applicable

## Appendix

Please insert table 1 here

**Table 1:** Search strategy in Pubmed; Search conducted on June 2021

Search number	Query	Results
5	(((adolescen* OR youth OR young adult OR young people OR child) AND (school-based tobacco prevention program* OR in-school tobacco prevention program* OR routine tobacco prevention program* OR standard tobacco control program* OR school health promotion)) AND (smok* OR tobacco use)) AND (low-middle income countr* OR LMIC* OR developing countr*)	380
4	school-based tobacco prevention program* OR in-school tobacco prevention program* OR routine tobacco prevention program* OR standard tobacco control program* OR school health promotion OR school health education	238,059
3	low-middle income countr* OR LMIC* OR developing countr*	272,348
2	smok* OR tobacco use	405,000
1	adolescen* OR youth OR young adult OR young people OR child	4,350,683

## Abbreviations

JBI: Joanna Briggs Institute
SUMARI: System for the Unified Management, Assessment, and Review of Information
GRADE: The Grading of Recommendations, Assessment, Development, and Evaluation
SoF: Summary of findings
LMICs: Low- and middle-income countries
HICs: High-income countries
GYTS: Global Youth Tobacco Survey
SHEP: School and education programme
PROSPERO: International Prospective Register of Systematic Reviews
PRISMA: Preferred Reporting Items for Systematic review and Meta-Analyses
PRISMA-P: Preferred Reporting Items for Systematic review and Meta-Analysis Protocols
Cochrane RevMan: Cochrane Review Manager
GRADEpro GDT: The Grading of Recommendations, Assessment, Development, and Evaluation Guideline Development Tool
